# A focus group study exploring dairy farmers’ perspectives of cull cow management in Ontario, Canada

**DOI:** 10.3389/fvets.2023.1189668

**Published:** 2023-06-06

**Authors:** Joanne Marshall, Derek B. Haley, David Kelton, Cynthia Miltenburg, Steven Roche, Todd Duffield

**Affiliations:** ^1^Department of Population Medicine, University of Guelph, ON, Canada; ^2^Campbell Centre for the Study of Animal Welfare, University of Guelph, Guelph, ON, Canada; ^3^Dairy at Guelph, University of Guelph, Guelph, ON, Canada; ^4^Ontario Ministry of Agriculture, Food and Rural Affairs, Guelph, ON, Canada; ^5^ACER Consulting Limited, Guelph, ON, Canada

**Keywords:** cull dairy cow, qualitative, focus groups, compromised cow, farm management

## Abstract

**Introduction:**

Maintaining the welfare of cull dairy cows from the farm to slaughter is an ongoing challenge for the dairy industry. Recent research suggests that some cull dairy cows within the marketing system are in physical states that are below regulatory standards, and further research is required to determine why these unfit cows are found throughout the journey to abattoirs. Since dairy farms are the origin of these cows, decision making by dairy farmers has been identified as key to preventing cull cows that are considered unfit for transport from entering the marketing system. The objectives of this study were to understand dairy farmers’ perspectives on their cull dairy cow management practices, recommendations and requirements of regulations, management tools, and welfare issues.

**Methods:**

Four focus groups with a total of 21 participants were each conducted virtually, video recorded, and transcribed verbatim, with dairy farmers from Ontario, Canada. A thematic analysis of focus group discussions was conducted utilizing deductive reasoning.

**Results:**

There were three themes identified including deciding to cull or not, management of cows being culled, and knowledge and perceptions of cull cow regulations. When making culling decisions, farmers utilize multiple sources of information including personal experiences and values and external referents like veterinarians, family members and other farmers. The welfare of their cows was a high priority but one that was often weighed against the financial outcomes of culling decisions. Finally, most participants considered recent regulatory changes for the management of cows before shipment to be of little importance on their farms.

**Discussion:**

In conclusion, the farmers from this study showed the diversity of considerations they make in culling decisions and the large contribution of animal productivity and economic factors. There was a general lack of knowledge of recent regulatory changes for the shipment of cull cows, and there is room for improving the uptake of new recommendations for culling only cows fit for transportation.

## Introduction

A regular and required practice in the dairy industry is the culling of cows, with roughly 30% of an average dairy herd being culled in North America and western Europe (1–3). In most cases, the turnover of cows in a herd allows for removal of poorly performing cows and the introduction of cows with higher economic potential into the milking herd. The higher economic potential of the replaced culled cows is a cumulative result of introducing cows with improved genetics and fewer health issues. Thus, the removal of cows from the herd is essential to the productivity of dairy farms. Farmer decisions in Canada are further influenced by the nature of the Canadian dairy industry; more specifically, in Canada, a supply management system is used to allocate volumes of milk produced at the farm and provincial-level. To meet delegated quota, farmers may be more or less likely to cull cows (4). For example, farmers will be disciplined (e.g., financially penalized) for over production, but sometimes farmers are financially incentivized to produce more milk in response to predicted market demand (5).

Dairy cull cows can largely vary in their condition at departure from the farm including being in a state of lactation or disease (6, 7). Although research on culled dairy cows is limited, research shows that this variation in clinical state extends to regions outside of north America (8, 9). In Canada, most cull cows are transported off the dairy farm to an auction facility, and from here they will be bought and transported to an abattoir for slaughter. A high proportion of compromised and even unfit cows have been sold at auctions, and these cows are at risk for considerable suffering (10). In Canada, a compromised cow is not supposed to be transported to assembly centers (e.g., auction facilities) and may only be transported directly to a destination for humane slaughter or veterinary care (11). Some signs of a compromised cow include being in peak lactation and will not be milked to prevent udder engorgement, any lameness (not classed as unfit), and exhibiting any signs of illness or injury limiting ability to withstand transport (11). The Canadian Food Inspection Agency (**CFIA**) describes an unfit cow as one that cannot be transported unless it is under the direction of a veterinarian for care, and some signs of an unfit animal include being non-ambulatory, having a fracture impeding mobility or causing pain, and having labored breathing (11). In August 2017 in Canada, 27, 41 and 73% of cull cows had unacceptable hock injuries, body condition score (BCS), and gaits, respectively (12). Cull cows experiencing welfare issues like pain and disease are less able to withstand the challenges of transport including interacting with other animals, fatigue, feed and water restrictions and thermal extremes (13).

In Canada, the average productive lifespan of a cow on a dairy farm is about 4 years (14). In the dairy industry, some common reasons for the removal of cows include infertility, poor production, mastitis, and lameness (i.e., abnormal locomotion) (1, 3). Oftentimes, cows are cited as being removed from a herd due to a singular reason, but numerous factors may lead to a cow being culled, which further increases the risk for deterioration of conditions during transport (15, 16). Historically, it has been reported that compromised dairy cattle and those unfit for transport arrived at slaughter plants with no significant disincentive to those profiting from the sale of the animal (10, 12, 17). This may be due to some farmers and veterinarians not having a clear understanding of what the market and the journey to slaughter plants may entail for cows (10, 18). However, there is an incentive to transport cows of poor fitness to avoid the cost of euthanasia (10). In Canada, the dairy industry has attempted to resolve welfare issues for cull dairy cows through the Dairy Farmers of Canada, an advocacy group for Canadian dairy farmers, introduction of the proAction initiative (19) and government regulations (11). Most recently, the CFIA, a regulatory agency of the Government of Canada, updated the Health of Animals Act with new regulations for transportation of animals. These new regulations have put further emphasis on preventing suffering for cull dairy cows (11). Finally, the dairy industry’s intersection with the beef sector makes total resolution of welfare issues for cull cows quite difficult for both industry and government regulators.

Although culling cows is inevitable and commonplace on dairy farms, the influence of economic, social, management, animal disease, and regulatory factors vary widely between farms (20). The diversity of factors that must be considered in a culling decision and varied adherence to recent updates to regulations for acceptable practices within the industry largely account for unfit or compromised cows entering marketing systems. Inaccessibility of alternative slaughter destinations cannot be discounted as influential factors in welfare issues for cull cows. The perspectives of dairy farmers on decision-making for culling cows is a knowledge gap that limits further strategy development for changing the poor welfare outcomes cull cows often endure. Therefore, the goal of this study was to investigate the perspectives of dairy farmers on management, recommendations and requirements of regulators, decision-making pertaining to cull cows, and welfare issues.

## Methods

### Study design

Dairy producer focus groups were used to identify factors that influence farmers’ management of cows being culled from Ontario dairy farms. This study was approved by the research ethics board of the University of Guelph (REB #21–09-012). The data collection took place between December 2021 and January 2022.

### Data collection

Four focus groups were conducted with a convenience sample of farmers who had indicated their interest in being contacted for further research participation from an earlier cull cow survey.

The 79 potential participants were recruited by an email sent out in December, 2021. Any dairy producer contacted was eligible for inclusion if they were actively a dairy farmer in Ontario and at least 18 years of age. For participation in the focus groups, participants were offered a $100 gift card for compensation. Participants were divided into focus groups based on their preference for the set dates and times. The number of participants per group ranged from 3 to 8 (21 participants in total). The focus groups were held virtually using the Microsoft Teams meeting platform. Consent forms were signed before the recorded discussions were held, and the participants were given at least 7 days to complete informed consent review and ask any questions of the principal investigator (T. D.). Each group was interviewed separately by the same single researcher, but in 1 focus group, a second researcher was present to observe the conduct of focus groups and learn about qualitative research and had no active participation in the conduct of the research.

Focus groups were facilitated by 1 female researcher (JM) from the University of Guelph. This facilitator had undergraduate and graduate education related to the dairy industry and approximately 10 years of experience working in the dairy industry. The practical experience ranged from several years of regularly milking on commercial dairy farms to assisting in completing research projects investigating dairy calf management. The researcher ensured qualitative methodology was applied appropriately through coursework and the review of methodology by co-authors throughout study design and conduct. Using gaps in research determined by a literature review, a previously conducted survey, and personal experiences, the question guide was designed by all the authors and was used for all the focus groups to ensure all topics of interest were covered. The facilitator was allowed to rephrase questions and add probing questions during the conversation to keep the discussion from stalling and on course. Focus groups were between 40 and 100 min (averaging 78 min) and consisted of 3 parts: introduction and engagement questions, and exploration questions.

Following an introduction, the focus group participants were asked exploratory questions about their decision making for culling cows, standard operating procedures, management practices, and views on recent regulations. The semi-structured question guide was used to initiate discussion on these specific topics, and secondary questions and probes were used when necessary to obtain additional information. The interview guide is included in Supplementary material (Supplementary File 1: https://doi.org/10.5683/SP3/P5KWYN).

### Analysis

**Figure 1:**
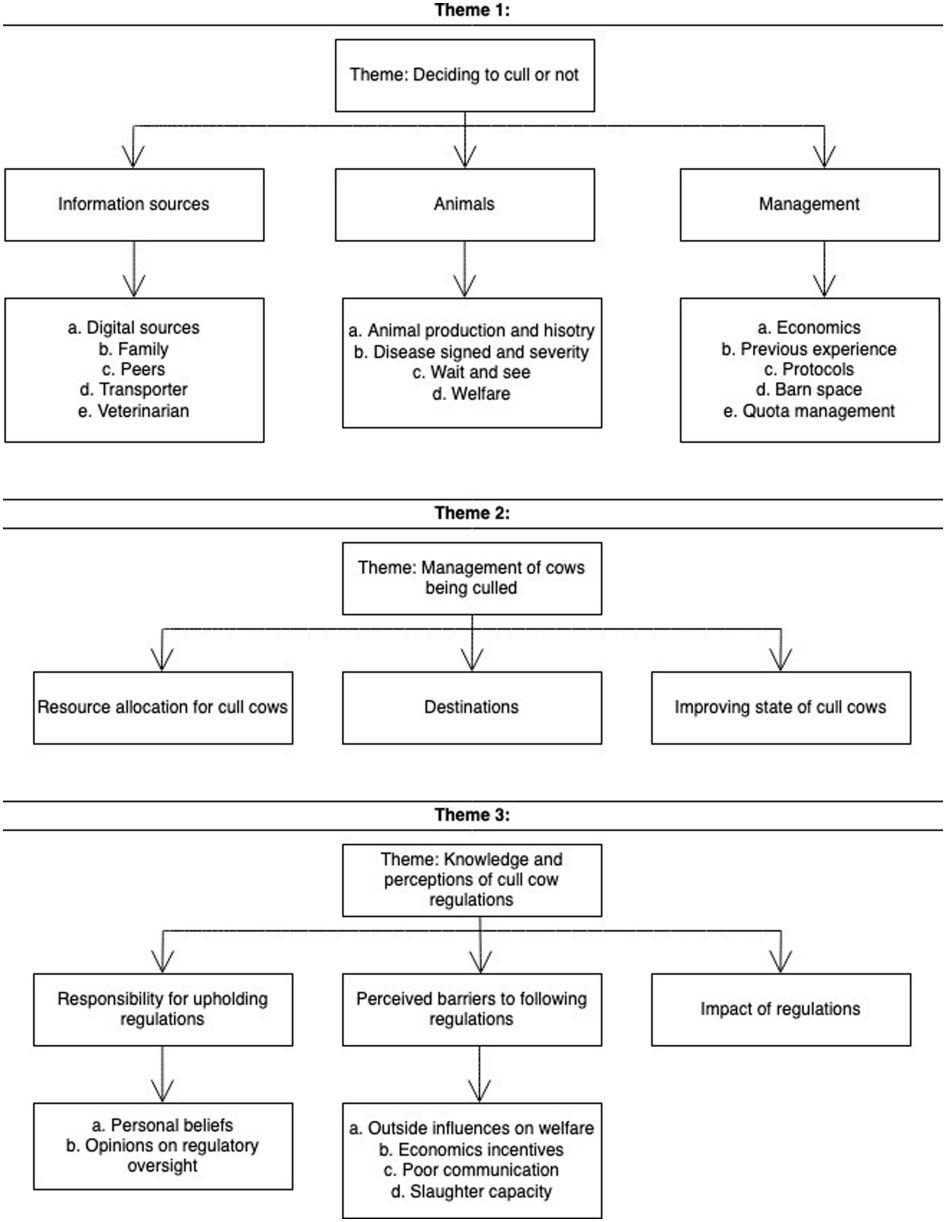
Thematic map of the analysis of data from the four focus groups of dairy farmers on cull cows, their management, and their opinions.

Focus group audio-recordings were transcribed manually verbatim by the first author (JM), and the same author coded and analyzed the transcripts. Thematic analysis was utilized following the methods of Braun and Clarke (21). The use of thematic analysis was selected given this methodology allowing for data to be encapsulate the results and potential to lead to unanticipated insights (22). Following the completion of the focus groups and familiarization by transcription, the researcher generated a data analysis plan with *a priori* codes using the method outline by DeCuir-Gunby et al. (23). Following a deductive approach, these codes were developed from existing theories and concepts into an initial codebook (23, 24); emergent themes and subthemes were identified leading to additional emergent codes being added to the codebook (23–25). The analysis process required systematic repeated examination of the raw data and reviewing of themes in an iterative process (21). Following coding, the relationships between codes were investigated and a thematic map was made to depict them (Figure 1). From a deductive standpoint, the relationships between themes and codes were informed by literature (15, 18, 26–28).

For reporting results, unique identifiers (e.g., A2) were assigned to individuals for quotes with the letter representing the focus group (A-D) and the number representing the participant within the group. To make quotes more easily interpreted within the research paper, the researcher performed condensation of the transcripts by removing verbal ticks (e.g., well, ah, um) and simplifying run-on and compound sentences. Square brackets (i.e., […]) were used to indicate when a quote was shortened or for when explanatory information was added to ensure meaning was made. When words like “um,” “ah” or repeated groups of short words that were used as a filler, pause, or hesitation were said, a dash (i.e., −) was used to indicate this in quotes. There are quotes reported throughout the results to illustrate key points made regarding the themes identified.

## Results

All 21 participants were all from Ontario, Canada. There was at least one participant from each region of Ontario (northern, eastern, southern, western); however, 16 participants identified their farm was located in western Ontario. Most participants were male (76%). All of the participants’ farms were family-owned and were farm owners and/or children of farm owners. Of those that indicated their farm’s barn style and milking system, 44% were tie stall, 37% were robot free stall, and 19% were parlor free stall. Finally, the average farm size of participants was 105 milking cows with a range of 53 to 470 cows.

### Key themes identified

There were three themes identified from the guided discussion with the participants. Theme 1: Deciding whether to cull a cow or not; Theme 2: Management of cows being culled; and Theme 3: Knowledge and perceptions of cull cow regulations. The analysis of data from the study is presented in Figure 1 as a thematic map.

### Theme 1: deciding to cull or not

#### Subtheme 1: information sources

Deciding whether or not to cull a cow from the herd requires farms consider cow-level and herd-level factors along with rules and their own personal values. Since this is a complex decision-making process, the choice to cull a cow, when to remove her from the herd, and the destination for a cow is often made with the consultation of various information sources.

##### Veterinarian

Participants frequently mentioned they were likely to contact their herd veterinarian regarding management decisions on their farms. For cull cows, they were especially likely to consult their veterinarian when they were unsure of the fitness of a potential cull cow for transportation, as participant [A5] commented: “*I consult with our vet quite a bit on a regular basis on cull cows...If I have a cow that is questionable, I do send him pictures, and he reviews them.*” Similarly, participants reported that veterinarians’ identification of cows with fertility problems lead to them listing her as a potential cull cow, as one participant said: “*When an animal - […] is, for instance, not fertile any more or whatever, [the] issue gets highlighted by the vet [as] not being an animal to keep long in the herd. We assign ‘do not breeds’ at that point.*”

##### Family

Some of the participants identified members of their family as being secondarily involved in culling decisions and cull cow management. While others commented members of their family were integral to these management decisions and actions, as said by the following participants [D5]: “*I am probably the main guy on the farm but also […] my mom. She’s probably involved more than anybody else.*”

##### Peers

Participants expressed that they received input from their peers in the dairy industry on management topics for their cows. For example, participant [D1] commented: “I *have a group chat with neighbors around here that are farmers. We all once a year-a couple times a year, we always have this big discussion[to] just try and have a discussion[or] have a talk about what we want to do for something like that [creating protocols]. Everyone just throws their couple of thoughts out there, and it’s sort of just like this [the focus group meeting]. Some of us are going to agree with it and some of us do not, and if we like it, we write it down ourselves. Everything is all open.*” Another participant commented about how a disagreement in opinion with a neighbor in management practices for culling relatively older cows was something that made him reconsider his management decisions: [A4] “*I was challenged a few years ago. I got a beef [farming] neighbor who - challenged me on shipping old cows, and in his opinion, if she is 8, 10, 12 years old there should be no auction destination for that cow. - Direct to slaughter would be the option. […] He’s of the belief that you euthanize that cow. […] It’s an [opinion on maintaining] animal welfare. I mean that’s where she is going to end up in her life, you know, and so do not put her through that last day of trauma. […] I do consider it - somewhat.*”

##### Transporter

Most participants commented they have had a transporter purchase at least for some of their cull cows. Farmers have discussions with these individuals, and some consider the opinions of livestock transporters in making culling decision, as indicated in the following statement: [A2] “*We’re all guilty of hanging onto those cows too long, and my trucker has made that comment before about the direct to slaughter cow I should’ve left - six months ago.*.” Participants frequently commented they expect a trucker to have the ability to properly assess cull cows’ fitness for transportation, as participant [D6] said: “*With our main trucker now, as soon as he takes her on his trailer, he wants - he chooses where it goes; that’s it. […] He chooses where they go. That’s how he wants to play the game -, and I have full trust in him - doing that.*”

#### Subtheme 2: animals

The decision to cull a cow from the herd is generally motivated by numerous factors. Specific animal cues that notify farmers that a cow should be culled that participants in this study cited were:

##### Animal production and history

Reproduction failure by cows was a reason commented as being a primary reason for cows to be flagged for potential removal from the herd and then culled from the participants farms. For example, participant [D8] commented: “*, I’ll have certain cows that maybe did not get pregnant fast enough, and I put them [on] like maybe a ‘do not breed’ list.*” Once culling for reproductive failures was discussed, participants were likely to also cite milk production decreases as being a reason for removing cows from their herd. As said by participant [C5]: “*Obviously, [we cull cows] if they are infertile or if they have dropped their milk so low that you know that they will not make it to a lactation without being too low [in milk production] and being dry too long. - Udder break down is another one; where […] they are too old then it [the udder]has broken down to the point where another lactation is not [going to] be viable.*”

##### Disease signs and severity

Participants also suggested there were conditions they regularly monitored that, when present, cued them to cull. For example, the following participant commented: [D8] “*Any cows that-that have tested positive for any sort of disease. - Disease like Prototheca or Staph [forms of mastitis] [will be culled].”*

##### Wait and see

For some conditions like high somatic cell counts or lameness, participants discussed waiting days before choosing whether to and when to cull a cow, and this was noted by some as being frequently used for cows they wanted to cull, but they felt were not yet fit for shipment. For example, one participant recalled: [A5] “*A lot of times I will – […]get her fixed up, and you know, wait two weeks - until she is good to go, and if she is good to go, then she will go to the stock yards.*”

##### Welfare

The welfare outcome of cull cows was discussed, and participants felt they performed practices for the welfare of their cows. When making culling decisions, participants indicated they preferred the option of perceived greatest welfare for the cows they culled. Like stated by participant [C5]: “*I spend a lot of time caring for my animals getting them ready to leave the farm in a [good] animal welfare state.*” Regarding the destinations for cull cows, participants commented some destinations resulted in better animal welfare and financial outcomes. For example, participant [C1] commented choosing transportation destinations other than auctions: “*We have started more doing direct sales[…] to like hobby farms […] By far, […] we know them well enough [that] we know they are getting well taken care of*..”

#### Subtheme 3: management

Despite many culling decisions being cited as motivated by singular cow-level factors, other factors often alter decisions and when they are made. The factors that were intrinsically related to the management of that farm that influenced culling decisions by participants included.

##### Economics

Participants frequently considered the potential economic consequences of their decisions for culling cows. The desire for optimal financial outcomes from culling decisions and numerous considerations for this were frequently brought up in the discussions, as expressed in the following quotes of participants discussing potential cull cows: [C3] “W*ell, if she looks skinny, we just do the math. How much does the surgery cost? How much does the medication cost? How much milk are we losing with her withdrawal periods. […] We just do the math on cost* versus *gain, and often, there is not a lot of gain on our farm on [compromised] cows.*”

##### Previous experience

When discussing how and when during a cow’s lifetime farmers identify cows to cull, participants frequently expressed that their previous experiences and knowledge of that was largely a contributor. This was most elaborated on by participant [D2]: “*When I first started farming, − it was hard for me to identify - which cows [were] starting to lose their […] level of health […]. Taking the time to identify cows sooner and try[ing] to move them out faster before they become a problem. […] I think experience has helped me there*.”

##### Protocols

When asked about protocols used for cull cows, participants frequently mentioned having a standard operating procedure (**SOP**) for assessing cows immediately before transportation [A1] “*We all have - shipping cow protocols for when she leaves. As far as checking treatment records, broken needles [broken needles that become embedded within an animal’s tissue without removal], making sure she has the appropriate tags, and she is identified as fit for transport […] that check list - that comes out.*” Of the farmers that had an SOP, all the farmers affirmed that they mainly had them because they were required to by industry regulations. Most participants reported not regularly referring to SOPs for cull cows, as commented by the following participants [B3]: “*Yea we have one [a standard operating procedure for assessing cull cow fitness] too, but I do not know what it says.*” Some farmers even said they did not have a written protocol for the shipment of cull cows, as one participant said [C5]: “*I will be honest it [a SOP for culling cows] is not written down.*”

##### Barn space

Some participants commented that the amount of barn space they had sometimes influenced culling decisions. During a discussion on the largest challenges farmers felt they face for cull cow management, one participant commented: [D3] “*I find - space, like physical space, does - play a factor in what you think you might do with a cow. […] I find - when you get into a space crunch, you have to make […] quick decisions sometimes.*”

#### Quota management

Some participants expressed that the amount of quota they had to meet impacted their likelihood to cull cows. Some farmers commented that quota and presence of incentives lead them to make delayed culling decisions. As said by the following participant: [C2] “*It [culling a cow] depends on are we in fall incentive, are we -, where are we on our quota? Are we filling our quota? […] If I am in fall incentive and [a] cow had an LDA [left displaced abomasum], I fix her and then I carry on. - If I picked it up pretty early, the prognosis is pretty good, she will probably recover and give me a few thousand dollars in milk, and then, I will send her properly on a sale to the sale ring - at the end of fall incentive.*”

### Theme 2: management of cows being culled

#### Subtheme 1: resource allocation for cull cows

Compared to financial investments, a lesser acknowledged resource in farming that was mentioned by one participant is time, which can be used to discuss and thoroughly consider some management choices. For example having the time to plan transportation destinations or provide extra care for cull cows: [D4] “*It always takes some time to get them in there [to local slaughter facilities]. It’s not like, you know, you can decide to ship them [there] this week […] You almost have to book it ahead of time.*”; [C5] “*I spend a lot of time caring for my animals getting them ready to leave the farm in a[n] animal welfare state.*” Sometimes a potential cull cow will be given fewer resources like treatment, feed, or milking frequencies, as one participant said: [A4] “*We just treat the cows as we do all the cows. The only thing I would say is that–maybe in the week ahead, we will drop the feed in the robots to get that grain reduction.*” This may be to prepare a cow for transportation or an economic-based decision. Therefore, just as much as participants commented they restrict some management practices for cull cows, participants reported providing small amounts of resources to cull cows.

#### Subtheme 2: destinations

Most farmers have choices for a potential destination to send cull cows including transportation directly to a slaughter facility, auction markets, or other dairy farms. Although, some participants pointed out they felt there has not been enough capacity at slaughter facilities for cull cows to be sent directly to slaughter, which participant [A1] commented on: “*In lots of cases, there is just not enough space to get these cows direct to slaughter, so I think as an industry it is really tough to make an improvement if we do not have the resources to send those cows quickly where they need to go.*” For cows producers felt were compromised, participants were likely to suggest the preferred route to slaughter would be ‘direct to slaughter’ (i.e., transportation from the farm directly to a slaughter facility); as one producer stated: [A4] “*[If] we do not think that animal is going to do well at the sale, [like] she is not walking properly, [we send her] direct to slaughter.*” When selling cows to transporters, some participants commented that the transporter made the decision for the destination: [D6] “*As soon as he takes her on his trailer, he wants-he chooses where it goes. That’s it.*”

#### Subtheme 3: improving state of cull cows

Farmers were asked if they employed any different management practices for animals identified to be culled. Although most farmers began their response to this with a statement that their cull cows were treated the same as the rest of the cows in their herd, most followed this comment by elaborating on some special management practice they conducted specifically for, at least, certain cull cows. As stated by the following participant: [B1] “*They get the same care as all the other milking cows [be]cause they are in the same stalls and everything. But then, − what I do closer to the date of shipping: I put them on first cut dry hay, like a lower energy feed, and then, I - skip milkings for three days. […] I also put them in a like pack [be]cause they are in tie stalls. […] then they are more likely to be mobile when they get on the truck.*”

### Theme 3: knowledge and perceptions of cull cow regulations

#### Subtheme 1: Responsibility for upholding regulations

##### Personal beliefs

Initially, all farmers expressed their responsibility for the fitness of cull cows for transportation and slaughter ended when the cow left their ownership, which was most often by a drover. As indicated by participants [C2]: “*Once it leaves the farm and goes on the truck, it is the trucker’s responsibility, and once it leaves the truck and goes to the sale ring, it is the sale ring’s responsibility. It is, in my mind, whoever has [financial] possession of the animal.*” In contrast, there were farmers that acknowledged their responsibility for factors that impact the quality of beef like broken needles and antibiotic treatment withdrawal times: [D6] “*Normally, you would think that at auction, once the animal is sold, it is no longer your - responsibility […].-When it comes down, you know, to say embedded needles or antibiotic residues, it, − the producer should be responsible for it. But if they have done their - best practice up to that point, it should be all- fair and - straight forward. So, it is pretty hard to take responsibility beyond the auction at that point.*”

##### Opinions on regulatory oversight

When regulatory standards for the condition of cull cows at markets and slaughter facilities are not met, inspectors may elect to euthanize a cow. Participants frequently mentioned they were unhappy with the circumstances surrounding a cull cow that had been identified as unfit, condemned, and euthanized at auctions. In more than one focus group, there were participants that expressed dissatisfaction with the communication from auction and slaughter facility personnel regarding cows that were eventually euthanized. One participant elaborated on a negative experience that resulted from a cow culled from his farm: [A4] “*[I had a] bad experience [that] involved like an investigation. They - interviewed my vet; they interviewed my trucker. I felt my reputation was being challenged, and - that was almost more damaging than the actual [financial] cost involved. […] I just felt raked over the coals.*”

Although there was ample discussion regarding dissatisfaction with regulations and their enforcement, there was acknowledgment by participants of the usefulness of regulations for encouraging “proactive” culling and betterment of animal welfare. As one participant commented: [C3] “T*hey [have] tighter restrictions on what you cannot and can ship. So, we just got more proactive on it, and I think, overall, it helped our farm to be honest. It places more emphasis shipping an animal in good health and not waiting too long if they are not in good health.*”

#### Subtheme 2: perceived barriers to following regulations

##### Outside influences on welfare

Despite regulations existing in Canada for the length of time cows may be transported before receiving feed, water, and rest, participants expressed concern for the welfare of cull cows experiencing lengthy transportation times before slaughter: [C3] “*We do not know actually how long an animal is going to be on transport until they get to their final destination*.” Along with the issue of distance and time traveled, some also expressed that those handling cull cows beyond their farm may affect cull cow welfare: farmer [C2] said, “D*oes it matter what we are doing and all the protocols we have? They mean nothing when that animal gets on the truck - they are at the mercy [of] whoever is going to handle them and how long it is going to take them to be [slaughtered]*.”

##### Economic incentives

Recent regulations within the Health of Animals Act implemented for cull cows require cows lactating at a high level of production be milked before transportation with an aim to limit the level of udder engorgement. Some farmers commented they felt there were barriers to their implementing the new rules for cull cow transportation on their farms. One farmer commented: [C2] “*I do have issues sometimes with the dairy ring sales. […] I’ve sold animals, I’ve milked them in the morning, and I sell them in the dairy ring and do the proper things, so the animal is in good condition for sale. But you do not get proper - you do not get a proper price at the sale because you did not udder the animal up. - I have real issue with not milking my animal before they go to a sale so that they can go on a truck for 12 to 14 h and go to somebody else’s place. [...] I do not like that philosophy, but you end up doing it so that you can get proper value for the animal.*”

##### Poor communication

Similarly, participants commented that they felt there was not enough communication between them and those involved in the sale of cull cows. They felt there was not enough communication regarding the sales price for cull cows, as reflected in the following statements: [C2] “*[I would like] a better way of tracking - why an animal sold, […] and better maybe a description or some tracking to know why did my fresh cow not sell well.*”; [C3]: “*The odd time you do have a condemned animal...They write down in ‘doctors’ language’ [using medical terminology] where it is so - [there] is no way to read what the actual reason was that the cow was condemned. I mean I would really like to know, so I can avoid that next time […] I wonder if it is done on purpose. Tell me what is wrong.*”

##### Slaughter capacity

The limitations for local abattoir space and number of cows able to be sent directly to slaughter was commented on frequently by participants. The dilemma farmers sometimes face was elaborated on by participant [A1]: “*Abattoirs spaces are certainly limited. But, it would be - I think it would be beneficial if there would be more opportunity to send cows to direct slaughter because let us face it, all of us have cows that [are compromised].*”

#### Subtheme 3: impact of regulations.

Some participants commented that they felt regulations were not effective in changing their management practices. One participant commented on their conflicted feelings toward new management practices required and feeling unsure about decision making around transporting a cull cow versus euthanizing on farm: [D4] “*I do struggle, when we have to get that [a captive bolt gun used as a stunning method before euthanasia] out for a[n otherwise] perfectly good cow. - With regulations that there are, we all have - cows that you have to put down. Where before, we did not have to do that. Sometimes, it’s a guess work.*” In a discussion about whether regulations for cull cows had impacted animal welfare, participants occasionally mentioned feeling overwhelmed trying to keep up, as the following participants said: [A3] “*I hate to say it, but we have not changed our cull cow practices. If anything, − it’s probably, I’ve probably paid less attention to it. […] It’s just trying to get everything into a day.*”

When recent regulatory changes for the shipment of cull cows were discussed, most of them acknowledged being familiar with some changes occurring, but in further discussion, many of the participants discussed actually not being fully aware of the changes to transportation regulations implemented in 2020. Some participants expressed feeling some changes were not fully necessary to be completed before they transported a cow off their farm. For instance, one farmer said: [A2] “*I was aware of them [recent changes to regulations] just because we have been reviewing the code lately so much. There wasn’t anything major I felt was going to affect me.*”

## Discussion

The perspectives, sources of information, and opinions of Canadian dairy farmers were explored regarding culling decisions. Our main findings were that farmers personal experience and perceived knowledge of best management practices were first to be considered when deciding to cull a cow, but individual animal’s histories, economics, and opinions of others were also commonly considered in culling decisions. Farmers were not aware of some requirements for culling cows like the required milking of heavily lactating cows for reduced udder engorgement (11). There was marked frustration by farmers regarding the functionality and communication with the current system for cull cows transported off the farm. Farmers were critical of the importance of new regulations for cull cows, and their personal values and current knowledge of requirements were identified as barriers to the uptake of practices according to regulations and use of management tools.

### Information sources

Farmers will consider external information sources and differing opinions to their own in making decisions they feel ambivalent about. As commented by participants, the herd veterinarian is a common source of information, which reflects reported involvement by veterinarians in culling decisions. In 2014, the USDA reported veterinarians were involved in culling decisions about 24% of the time in the U.S. and veterinarians designed protocols specifically for culling decisions 7% of the time. In Canada, the National Farmed Animal Health and Welfare Council reported herd veterinarians need to play an active role in guiding farmers on determining the fitness of cows for transport (29). With culling decisions being increasingly scrutinized among many other aspects of dairy farming, farmers have been seeking input more often and from additional sources. This reflects findings from Sumner et al. (30), which reported that farmers who were unsure about their assessment of a calf were introspective about their management decisions. However, some farmers did comment on their desire to continue with the previous level of consideration for the fitness of cows for transportation. This reflects the findings of Te Velde et al. (31) that some farmers who were unsure of their management decisions fall back to the status quo of previous practices they have used. From these contrasting findings, we may conclude that the level of consideration farmers have toward managing cull animals and seeking further information about new recommended practices differs by personal values (11). These findings indicate there continues to be a need for reporting to farmers of the importance of improving the welfare of cull cows being transported. Since farmers highly value information from veterinarians, increasing the communication between farmers and veterinarians is a key strategy for addressing knowledge, transport, and translation of appropriate cull cow management practices.

### Economic considerations

As farming is a business, farmers commenting on possible economic considerations in culling decisions was an anticipated result. As McInerney (32) pointed out, economic outcomes are significant to decisions about utilizing animals, but farmers sometimes provide for animals’ welfare beyond financial justification. Similarly reflected in our results, the financial outcome of management decisions was often first cited as influential to culling decisions. Although the possible financial outcomes from culling decisions were highly influential, participants readily commented that they also placed a high value on the welfare of animals. They expressed that along with animal welfare being a moral obligation for animals they felt the welfare of cows from their farm was a representation of their integrity, which is consistent with a previous finding by Croyle et al. (26). Oftentimes, participants followed their comments on the monetary outcomes of cull cow management decisions with an additional statement of their need to also consider welfare outcomes for a cow, which stressed the ethical obligation they have felt making management decisions for their cows.

### Cues to cull

Farmers commented that after noting certain cues to cull a cow, often production and disease related, they would often wait and monitor cows for signs of disease before making the final decision for culling. Therefore, farmers continue to struggle to make culling decisions for both optimal economic and welfare outcomes. With research determining cull cows are at risk for deterioration in condition between being placed on a culling list to arriving at an abattoir, early or proactive culling has been increasingly recommended (17, 33, 34). From our results, there continues to be room for improvement in making timely culling decisions on farms, aimed at optimal cull cow welfare.

### Culling reasons

The most common reason cited for the removal of cows from the participants’ herds was reproductive failure, which was followed by low milk production. These responses closely reflect reported reasons for culling cows (1, 35). In Canada, the top three cited primary reasons for culling cows from dairy farms were reproduction, mastitis, and feet and leg problems (1). In the USA, 21, 21, and 17% of cows were culled for infertility, poor production, and mastitis, respectively (35). In addition to these production and disease related reasons for culling, farmers indicated their previous experiences, their barn capacity, and farm goals were also common contributors to a culling decision. In Canada, dairy production is supply managed and federally regulated (36). During seasons of increased demand for dairy products in Canada, farmers are financially incentivized to produce more milk (5). The industry’s high supply of young cows and heifers has potentially led to some farms lacking space in their barns, which as participants noted, results in a need to increase the rate of culling cows. In contrast, participants indicated the presence of high production incentives lead to them decreasing the number of cows culled during the fall to winter seasons. As the dairy industry is known to be impacted by seasonality, farmers’ considerations of the impact of supply and demand periods when making culling decisions was consistent with culling trends (27).

### Fitness assessment for transportation

Even for cows sent to slaughter being healthy and physically fit, the duration and quality of the journey can represent a significant challenge (13). For cull cows that are weak, diseased, or injured, the challenges of transport can result in severe welfare issues (37). A cull cow may experience severe pain, fatigue, and sickness resulting from the stressors of transport including loading and unloading from vehicles, interacting with other animals, maintaining a standing position, and restrictions of feed and water (13). Farmers reported confusion in deciding whether a cow will be considered fit at markets following transportation reflects this. The lack of clarity on how to approach managing cows once they were identified to be culled indicates the need for producer education, especially related to the need for preventing udder engorgement (38).

### Regulations

Since 2020, dairy farms in Canada are required to have a written SOP for the shipment of animals (19). This protocol must be documented and contain all the information required for the shipment of cattle that are fit for transport while meeting federal and provincial regulations (19). Although cull cow related protocols have been recently required on farms, our results indicated that farmers have not been consulting them, thus have had little effect in changing the decisions of farmers or achieving the industry’s goals for cull cow management. In this study, most farmers indicated they had very little knowledge of the contents of their farm’s written protocols. As other studies have indicated, changes in behavior must be motivated by pursuit of a goal, and as this study indicated, most farmers were in a phase of deliberation regarding the need for specific management changes for cull cows, even though some actions were already enforced on farms (39–41).

By reducing the number and frequency of milking for lactating animals, milk production decreases, thus preventing painful udder engorgement following long periods of time without milking. According to our results, farmers are not likely to implement management practices to prevent udder engorgement. This result is consistent with reports of culled dairy cows within the marketing system and arriving at slaughter in North America at risk for udder engorgement (42, 43). However, these studies were conducted before Canada’s new transport regulations were put in place. The new regulations implemented prohibit heavily lactating animals from being shipped to destinations that will result in a high likelihood for udder engorgement. New studies on the prevalence of udder engorgement and other restricted conditions for culled dairy cows are necessary to evaluate the effect of the new regulations.

Farmers have a responsibility to follow the regulations put into place by their regulatory groups and the government. Following regulatory changes, the uptake of new management practices has been required for farmers transporting cows off farm (19, 44). As of 2020 in Canada, regulations require lactating animals must be milked sufficiently to prevent mammary engorgement (11, 44). To comply with this outcome-based regulation, lactating animals must be milked throughout the journey or not shipped until their milk production has decreased and the risk of suffering is reduced. From this study, farmers showed they were not considering this requirement as necessary or important as a practice on their farms. Further requirements under proAction should be implemented to reinforce and emphasize the lesser considered requirements for the management of cull dairy cows like that for heavily lactating animals.

### Responsibility

A common complaint from farmers was the lack of scrutiny toward other stakeholders in the cull cow industry. Handling is a major cause of stress for cattle during transport, and the possibility for cull cows to be handled by several people is high (46). Farmers pointed out that cull cows can spend lengthy proportions of time at auction markets, and that these are places for potential cull cow welfare improvement. As Edwards-Callaway et al. (10) commented, all stakeholders from those involved in livestock markets, transportation, and the slaughter process have a responsibility to protect the welfare of dairy cattle. To ensure this, inspections of cattle at markets by certified personnel exist. Currently, Ontario is the only province in Canada that has mandatory government inspection at livestock auction markets, which are conducted by government appointed veterinarians and works to ensure fitness of cattle within the marketing system (12). However, the issue of compromised and unfit cows within the marketing process still continues, indicating a need for cull cow welfare to be further addressed by the industry.

Minimizing stress and pain for cull cows is a responsibility of all the industry stakeholders, but as these results highlight, changes in ownership and custody of cull cows during the marketing process continues to be a major challenge for the maintenance of the welfare of cull dairy cows (10).

### Communication

Following a cull cow being declared unfit for slaughter either at auction or a slaughter facility, there were participants that expressed dissatisfaction with the amount of communication and feedback given. In multiple focus group meetings, there were complaints about the degree of scrutiny veterinary inspectors had toward the condition of some cull cows including cows being euthanized on-site at either auction or slaughter. In the event a culled cow is declared unfit along the journey to slaughter, even when the level of repercussion culminates in an on-farm investigation like mentioned by one participant, farmers should be treated with respect while investigating if best practices were followed along the custody chain. There is great room for improvement in communication between farmers and regulatory enforcers. With feedback to the farm of origin from groups involved in the journey of a cull cow, farmers may be better equipped to align their management practices with regulations. This will likely lead to improvement in management decisions by farmers and may even allow for some cows to avoid being euthanized and instead be slaughtered as intended.

### Perceived barriers for cull cow management

Farmers perceived the cost of implementing some required and recommended management practices for cull cows as a barrier to improving their fitness for transport. This is similar to other studies investigating farmers uptake of other industry requirements and recommendations like decreasing antimicrobial use (46) or Johne’s disease control (28). Like many businesses, time is a precious resource for the betterment of management and thus increasing the financial viability of the business. A commonly cited management practice for the betterment of culling cows at a rate for economic success and improved cow welfare is “proactive” culling. Even one participant specifically cited the thorough discussion of culling decisions on their farm as resulting in their ability to be more selective in which cows they culled; meaning the farmers consistently allocating the resource of time to culling decisions will be able to make those decisions earlier in a cow’s life. As numerous researchers have reported, culling cows before they experience diseases (e.g., lameness and mastitis) that affect their productivity and condition, allows for more financial gain for the farm (2, 12, 47). Therefore, further emphasis for allocating resources to making earlier culling decisions are necessary to help reduce cull cows of poor fitness within the marketing system.

### Slaughter capacity

A considerable proportion of cull dairy cows are sent directly to slaughter, which minimizes transport time, numerous stressors, and the total time to slaughter (10). Participants routinely commented that they send cull cows directly to slaughter facilities, and that they would like to send even more cows to slaughter this way. This reflects the consensus of industry members that more opportunities for local slaughter need to be identified (29). As regulations regarding the fitness of cull dairy cows have become stricter, the demand for opportunities to send cows directly to slaughter facilities increases. Furthermore, with increased scrutiny from stakeholders toward the fitness of cull cows within the marketing system, farmers are more likely to be inclined to send a higher proportion of their cull cows to local slaughter facilities. Thus, the industry should explore the availability of local slaughter options for cull dairy cows. In addition, farmers continued improvement in the fitness for transport of their cull cows may assist in mitigating the demand for local slaughter by allowing more fit cows into the marketing system.

### Cow price

In conjunction with this, farmers expressed they felt they did not always receive proper or fair compensation for their cull cows once they were slaughtered. Cull cows can represent a large income for farms, especially when assessing the profitability of an individual cow over her lifetime; therefore, farmers regard for price was an anticipated result (10, 27). The desire for more compensation for cull cows in part contradicts a desire to shoulder less of the responsibility for the fitness of these animals at slaughter. Additionally, farmers commented viewing buyers that transport cows from their farm as being responsible for maintaining animal welfare off the farm. The transporter does have responsibility for maintaining the welfare of cull cows, and in the United States, a cattle transporter training program includes sections on fitness for transport (48). However, in expecting transporters to shoulder much of the responsibility for the welfare of cull cows, farmers also have a responsibility to assess fitness for transport and ensure a transporter is informed of the needs of each cow, particularly with respect to compromised cows.

### Limitations

There are a number of limitations to this research that must be considered in the interpretation of the findings reported. First, qualitative research seeks to understand the nature of phenomena, and does not allow quantifiable measures or the inferences from quantitative methods (49). There were four focus group meetings conducted, and these were limited to our population of interest, which were dairy farmers in Ontario. Due to the COVID-19 pandemic restrictions, these meetings were conducted virtually, which may have impacted the likelihood participants attended or their comfort while participating. There are other stakeholders and contributors to the journey of culled dairy cows from farms to slaughter facilities that make important contributions to their animal welfare. Due to financial and time constraints, we were limited in the number of meetings conducted, and without these, focus groups would ideally have been conducted until data saturation and with other industry groups (e.g., contracted transporters) (50, 51). Using one research to code and analyze the transcripts may have weakened the reliability of the research results. Although there were efforts to reach mutual respect among participants and moderator, there may have been individuals that were reluctant to share their true opinions and issues with respect to culling cows (52, 53). Finally, the sampling for this research makes it susceptible to self-selection bias of participants with strong opinions or interest in the discourse of cull cows being more likely to participate and be more vocal while participating in focus groups.

## Conclusion

In conclusion, Ontario dairy farmers’ perspectives of cull dairy cows are influenced by economic factors along with social, regulatory, and animal-related factors. Individual’s own experiences were most central to their culling decisions, frequently with influence from veterinarians. Barriers to improving cull cow management for regulatory compliance and animal welfare were mainly adherence to traditional management practices and a lack of knowledge of best practice for this group of animals. Due to farmers’ low perceived responsibility for cull cow welfare beyond their farm in the journey to slaughter, information and communication through the major points of a cull cows’ journey may be a useful element to change behavior. Future research should focus on quantifying the outcomes of bettering on-farm cull cow management and assessing changes in management and cull cow welfare following education of farmers.

## Data availability statement

The datasets presented in this article are not readily available due to the sensitivity of the information within the focus groups, but some excerpts are included in this article. The full transcripts being shared were not consented to by participants. Requests to access the datasets should be directed to JM (jmarsh12@uoguelph.ca).

## Ethics statement

The studies involving human participants were reviewed and approved by University of Guelph. The patients/participants provided their written informed consent to participate in this study.

## Author contributions

JM contributed to recruitment, data collection, analysis, and writing the first draft of the manuscript. The interview guide was designed by JM and reviewed by all the authors. All the authors contributed to revisions of the manuscript and approved the submitted version.

## Funding

This project was funded by the Ontario Ministry of Food and Rural Affairs through the Agri-Food Innovation Alliance. Many thanks to TD for working to acquire this funding.

## Conflict of interest

SR is employed by ACER Consulting Limited.

The remaining authors declare that the research was conducted in the absence of any commercial or financial relationships that could be construed as a potential conflict of interest.

## Publisher’s note

All claims expressed in this article are solely those of the authors and do not necessarily represent those of their affiliated organizations, or those of the publisher, the editors and the reviewers. Any product that may be evaluated in this article, or claim that may be made by its manufacturer, is not guaranteed or endorsed by the publisher.
