# Supplementary material for: A focus group study exploring dairy farmers’ perspectives of cull cow management in Ontario, Canada
Source: Front Vet Sci. 2023 Jun 6;10:1189668. doi: 10.3389/fvets.2023.1189668 (PMC10279770; doi:10.3389/fvets.2023.1189668)
Supplement: Supplementary file 1 [file Data_Sheet_1.pdf]

# SEMI-STRUCTURED DISCUSSION GUIDE

## PART 1) Introductions and Engagement Questions (10min)

Before everyone arrives, the moderator participates in purposeful small talk to attempt to establish a warm and friendly atmosphere.

Moderator introduces themself including name, education, and background (i.e., hometown, general experience in the dairy industry). Briefly explain the moderator's (e.g., moderator will be posing questions, guiding discussion, but not inserting their own opinions on topics) at the start of the focus group. Although everyone has reviewed and signed informed consent forms, reiterate freedom to decline to answer any questions, anonymity of the discussion and following publications, and that the meeting is being recorded.

*Review the goals of the focus group:*

The aim of this research is to explore Ontario dairy farmers':

1) thoughts on cull cow management regulations, recommended management practices, standard operating procedures, and welfare issues

2) improve or develop educational and decision-making tools for managing cull dairy cows

*Introductions:*

Ask for everyone to introduce themselves with their name, major role on their farm, and their experiences with harvest this fall.

## PART 2) Exploration Questions (>=35min)

*a) Opening questions and training (10-20min)*

Please describe the care of cows identified for potential removal from your herd to their leaving the farm (I.e., specifically, how is the care of potential cull cows the same or different to cows just about to be culled from the farm?).

- Follow-up: Can you tell us a little bit about any training of personnel for the care of cull cows on your farm?
    - Probe: Are there any differences in the way people are trained to care for cull cows with differing conditions?
- Follow-up: What primary indicators do you use to identify a cow as being compromised or unfit for transport?

*b) Cull cow standard operating procedures (10-20min)*

Please describe any protocols for cull cow management on your operation.

- [Probe when relevant ensuring each of the below are discussed]:
    - Factors assessed

    - Timing of use
    - Regularity of use
    - Classification as SOP
- Follow-up: From what sources did you receive input to create or update a cull cow standard operating procedure?
    - Probe: Who or what do you trust most for information on cull cow management, and why?
- Follow-up: What or who was most helpful in creating your most recent cull cow protocol?
    - Probe: Can you elaborate on the advantages or disadvantages of using a standard operating procedure when managing cull cows?

*c) Cull cow management (10-20min)*

In your opinion, what are the most important management practices for optimizing cull cow care on your farm to ensure cows are fit for transport?

What are the biggest challenges you face regarding cull cow care?

- Follow-up: What would help you overcome these challenges?

Who do you feel should be responsible for cull cow welfare throughout the journey to slaughter?

In your opinion, what are the major issues in the management of cull cows today for farmers other than yourself?

Please explain any tools you use (or have used) to address any management questions you have for cull cows:

Follow-up: If you use (or have used) any tools, what are the advantages of the tools you use most?

Follow-up: What are the disadvantages to the tools you have used?

What recommendations do you have for future cull cow management tools?

*d) Cull cow regulations (5-10min)*

Are you aware of the changes that were made to the Health of Animals Act published in February of 2020 regarding cull cows, and if so, how do you think cull cow management has (or has not) been altered by these changes?

Follow-up: How do you feel the changes made to the Health of Animals Act have altered the wellbeing of culled cows, if at all?

Follow-up: Were there any changes made that you disagree with?

**PART 3: Cull Cow Case Studies (10-20min)**

*Scenario 1) [A compromised animal fit to send directly to slaughter] (5-10min)*

Following a regular visit from your veterinarian this morning, you have a cow that was diagnosed with a left displaced abomasum. She is early in her lactation with a body condition score below 2. You previously planned to cull her at the end of this lactation due to her reproductive failure, so you do not want to pay for her to have surgery. It is a Friday, and you know the sales yard will not be having an auction until Tuesday. Someone is available to transport her today and otherwise will not be available to transport her until next week. If she is on your farm, what do you do with her and why?

*Scenario 2) [An unfit animal for transport] (5-10min)*

You have a four-year-old cow with a body condition score of 3.5. She had watery milk with some flakes that indicated mastitis in her front left quarter but without noticeable swelling this morning. While walking, she arches her back mildly and reluctantly bares weight on her back right leg. She also shows signs of joint stiffness but is not impeded in her freedom of movement. She had mastitis in previous lactations that resulted in lower milk production compared to the average of the herd, and thus, she is on the farm's cull cow list for replacement with a potentially higher producing animal to meet your quota. If she is on your farm, what do you do with her and why?

**PART 4: Exit question and conclusion (10-20 minutes)**

Is there anything we have not discussed today that you think we should have?

*End discussion*

Thank everyone for their participation, remind them when to expect feedback and incentives, and say goodbyes.

*Exit email* (within 24 hours following focus group)

Thank participants for participation and send participatory incentives.

*Post focus groups*

- Describe the setting
- Describe the group
- Emotional tone of the group
- Any difficulties or challenges
- Any unexpected experiences
- General reflection and feeling toward experience
