# Supplementary material for: A focus group study exploring dairy farmers’ perspectives of cull cow management in Ontario, Canada
Source: Front Vet Sci. 2023 Jun 6;10:1189668. doi: 10.3389/fvets.2023.1189668 (PMC10279770; doi:10.3389/fvets.2023.1189668)
Supplement: Supplementary file 2 [file Data_Sheet_2.pdf]

DATA ANALYSIS PLAN AND CODEBOOK

Objectives

To investigate some Ontario dairy farmers' motivations and attitudes for the management of cull cows, and their perceptions regarding required management practices in the dairy industry for cull cows.

Inclusion Criteria

These focus groups only included individuals actively working as dairy producers in Ontario, Canada. The participants were recruited through a previously administered survey following their indicating interest in participating in further research on the subject of cull dairy cows.

General Data Analysis Plan:

For focus groups, data analysis required writing of transcripts, which were manually written verbatim. The data from the focus groups are composed entirely of text, thus requires qualitative evaluation. For this qualitative analysis, the text will be analyzed using thematic analysis. Furthermore, a deductive and semantic approach will largely be taken for this data analysis. However, some latent interpretations of the data and discussion will be explored by the researcher.

Firstly, the researcher will familiarize themselves with the data through the transcription process. Based on first impressions from the first draft of the transcripts, the research will create codes to capture both semantic and latent data a priori to further analysis. These transcripts will be reviewed for data immersion prior to beginning further analysis. Following this, the software program NVivo will be utilized to complete further analysis. Using the software, the research will conduct analysis of the transcripts by first constructing a table of general codes from the familiarization process and then openly code the responses into a coded table. During open coding, emergent codes will be created as addenda to a priori codes previously identified that may not sufficiently capture the data. Next, the researcher will identify the common themes from these codes, and then review and further define the final themes that emerge as most important and frequent within the responses.

To make quotes more easily interpreted within the research paper, the researcher will perform condensation of the transcripts by removing verbal ticks (e.g., well, ah, um) and simplifying run-on and compound sentences.

Themes, Subthemes, and Codes:

Codes: A Priori

Semantic codes

Name of Code	Code Description	Examples
Discussion topic related		
Animal production	A cow's quantity and quality of milk production	A cow not being pregnant by 120 days into a lactation

	A cow's reproductive success/failure	
Disease signs and severity	Systemic symptoms of illnesses and specific diseases mentioned as important to deciding and animal's future in the herd	Signs: Fever, pain, inflammation<br>Severity: depth of ulcers, amount of weight loss
Economics	Allocation, distribution, and utilization of financial resources	The cost of treating a cow for a disease<br>The earnings made from selling an animal
Individual animal history	Information about the cow's health and productivity	Quantity of milk in the last lactation, number of inseminations for previous pregnancies, current and ongoing diseases
Information source	Sources of information including individuals and discourses about cull cow management	The herd veterinarian, publications received in the mail from industry groups, online forums
Family	Family members roles in the decisions related to health and management decisions for cull cows	Spouse, children, uncle
Peers	Fellow farmers that are not family members contribute to management decisions including culling	Neighbours, members of industry groups
Previous experience	A farmer's previous experiences with the outcomes from reasons to decide to remove a cow from their farm	Receiving good financial compensation for cows without clinical signs of disease
Protocols	Comments about the role of protocols in the decision of removing cows from their farms	Having a standard operating procedure for assessment of cows for transportation
Regulations	Comments about the regulations impacting cull dairy cows historically, currently, and in the future	Seeing current regulations as being too difficult to adhere to
Reasons for culling	Specific factors cited as being a reason to remove a cow from the lactating herd	Low milk production, fertility, temperment
Slaughter capacity	Comments on the accessibility of slaughter facilities for cull dairy cows	Having limited access to timely local slaughter of cull cows

Transport availability	The availability of hired transporters or buyers of cull cows to quickly remove cull cows from the farm	Expressing transporters are readily available to transport cows off farm
Wait and see	A passive method of management of animals where an act is withheld for a variable period	Antimicrobial treatment of mastitis is withheld for days under the expectation of spontaneous clinical improvement
Interpersonal connections		
Agreement	Reinforcing another participant's statements	
Disagreement	Disputing another participant's statements	
Support	Sympathizing with another participant's statements	

Latent codes

**Name of Code**	**Code Description**	**Examples**
Active listening	Participants fully concentrating on what was being said	Eyes on the screen<br>Leaning forward
Agreement	Reinforcing another participant's statements	Head nodding<br>Mouthing yes<br>Using words of agreement like "I agree" or "Absolutely"
Disagreement	Disputing another participant's statements	Head shakes no<br>Stating words that express disagreement like "I disagree" or "Not necessarily"
Sarcasm	A comment made to humor or develop social bonds, or to imply displeasure with a topic.	Describing a disorganized person as being "very on top of things"
Lack of confidence	Indicating through patterns of speech or words used unsureness or unassertiveness	Using verbal ticks like um and uh, false starting sentences, and having regular pauses while speaking

Themes, Subthemes and Codes: A Priori and Emergent

**Semantic Themes, Subthemes, and Codes**	**Description**	**Examples**
Theme: Deciding to cull or not		
Subtheme: Information sources		
Veterinarian	A person qualified by a veterinary college to treat diseased or injured animals	The herd veterinarian or other bovine veterinarian.

Family	Family members roles in the decisions related to health and management decisions for cull cows	Spouse, children, uncle
Peers	Fellow farmers that are not family members contribute to management decisions including culling	Neighbours, members of industry groups
Transporter	The individual(s) that transport cows off their farm of origin	Hired person or farm personnel that transports cows
Subtheme: Moderators		
Economics	Allocation, distribution, and utilization of financial resources	The cost of treating a cow for a disease<br>The earnings made from selling an animal
Wait and see	A passive method of management of animals where an act is withheld for a variable period	Antimicrobial treatment of mastitis is withheld for days under the expectation of spontaneous clinical improvement
Welfare	Making culling decisions with the welfare outcome of individual cows in mind	Removing a cow from the milking herd due to welfare considerations for the affective state of the cow
Subtheme: Cues to cull		
Animal production and history	A cow's quantity and quality of milk production<br>A cow's reproductive success/failure	A cow not being pregnant by 120 days into a lactation; Quantity of milk in the last lactation, number of inseminations for previous pregnancies, current and ongoing diseases
Disease signs and severity	Systemic symptoms of illnesses and specific diseases mentioned as important to deciding and animal's future in the herd	Signs: Fever, pain, inflammation<br>Severity: depth of ulcers, amount of weight loss
Previous experience	A farmer's previous experiences with the outcomes from reasons to decide to remove a cow from their farm	Receiving good financial compensation for cows without clinical signs of disease
Protocols	Comments about the role of protocols in the decision of removing cows from their farms	Having a standard operating procedure for assessment of cows for transportation
Barn space	Comments about the need to only keep highest producing cows due	Having to remove cows (that are oftentimes older and

	to limitations in physical space in a cow housing facility	having more health issues) to make room for first lactation cows
Quota management	Comments on the need to either increase or decrease milk quantity or quality due to the quota management system	Having to reduce the number of cows producing low quality milk to better meet quota requirements
Theme: Management of cows being culled		
Subtheme: Resource allocation for cull cows		
Subtheme: Destinations		
Subtheme: Improving state of cull cows		
Theme: Knowledge and perceptions of cull cow regulations		
Subtheme: Responsibility for upholding regulations		
Personal beliefs	Ideas regarding responsibility for cull cows attributable to religious or personal beliefs, and past experiences	Commenting on the welfare of a cow being the responsibility of the person she is owned by
Opinions on regulatory oversight	Comments on the enforcement of regulations for cull dairy cows	Expressing satisfaction with current regulatory enforcement actions
Subtheme: Perceived barrier to following regulations		
Outside influences on welfare	Discussion regarding off-farm or other individuals' choices and associated alteration of cull cow welfare	Expressed concern for poor management at transportation destinations
Economic incentives	Comments on financial costs to following requirements	Having to spend more on labor to prepare a cow for transportation
Poor communication	Comments regarding the amount of and quality of communication between individuals and groups handling or managing cull cows	Not receiving notice of the poor condition of a cow before euthanasia after transportation off farm
Slaughter capacity	Discussion of the amount of available space for cull dairy cows at slaughter facilities	Low access to timely access to local slaughter facilities
Subtheme: Impact of regulations		

**Latent Codes**	**Description**	**Example**
Active listening	Participants fully concentrating on what was being said	Eyes on the screen and leaning forward
Impoliteness	Rudeness to cause social conflict or to communicate antipathy for a person or concept	Interrupting an individual speaking<br>Directly questioning another's idea to a point beyond disagreement

Lack of confidence	Indicating through patterns of speech or words used unsureness or unassertiveness	Using verbal ticks like um and uh, false starting sentences, and having regular pauses while speaking
Sarcasm	A comment made to humor or develop social bonds or to imply displeasure with a topic	Describing a disorganized person as being "very on top of things"
Codes for transitions between speakers		
Questions	Between moderator and participants	
Answers	Among participants	
Implicit continuation	Maintains the sample topic without saying so	
Explicit continuation	Maintains the same topic with an overt statement	
Change in topic by introduction of a new topic	Implicitly or explicitly introduced a new topic that shifts the content of discussion	
Change in topic by expansion	Implicitly or explicitly shares new aspects of an existing topic	
Change in topic by differentiation	Implicitly or explicitly compares different parts of a topic	
Codes for interpersonal connections		
Agreement	Reinforcing another participant's statements	Head nodes yes<br>Statements of agreement like "Yes", "I do that too", "I agree with that"
Disagreement	Disputing another participant's statements	Head shakes no<br>Statements expressing disagreement like "I disagree" or "Not necessarily"
Support	Empathizing with another participant's statements and distinctly supporting another beyond agreement	
